# Characterizing Livestock Markets, Primary Diseases, and Key Management Practices Along the Livestock Supply Chain in Cameroon

**DOI:** 10.3389/fvets.2019.00101

**Published:** 2019-04-10

**Authors:** Paolo Motta, Thibaud Porphyre, Ian G. Handel, Saidou M. Hamman, Victor Ngu Ngwa, Vincent N. Tanya, Kenton L. Morgan, B. Mark de C. Bronsvoort

**Affiliations:** ^1^The Roslin Institute, Royal (Dick) School of Veterinary Studies, The University of Edinburgh, Easter Bush, Midlothian, United Kingdom; ^2^Food and Agriculture Organization (FAO), Animal Production and Health Division, Rome, Italy; ^3^Royal (Dick) School of Veterinary Studies, The University of Edinburgh, Easter Bush, Midlothian, United Kingdom; ^4^Institute of Agricultural Research for Development, Regional Centre of Wakwa, Ngaoundere, Cameroon; ^5^School of Veterinary Medicine and Sciences, University of Ngaoundere, Ngaoundere, Cameroon; ^6^Cameroon Academy of Sciences, Yaoundé, Cameroon; ^7^Institute of Ageing and Chronic Disease and School of Veterinary Science, University of Liverpool, Leahurst, Neston, United Kingdom

**Keywords:** livestock markets, infectious diseases, management practices, Cameroon, supply chain

## Abstract

Live animal markets are common hotspots for the dispersal of multiple infectious diseases in various production systems globally. In Cameroon livestock trade occurs predominantly via a system of livestock markets. Improving the understanding of the risks associated with livestock trade systems and markets is, therefore, key to design targeted and evidence-based interventions. In the current study, official transaction records for a 12-month period were collected from 62 livestock markets across Central and Southern Cameroon, in combination with a questionnaire-based survey with the livestock markets stakeholders. The available information collected at these markets was used to characterize their structural and functional organization. Based on trade volume, cattle price and the intensity of stakeholder attendance, four main classes of livestock markets were identified. Despite an evident hierarchical structure of the system, a relatively limited pool of infectious diseases was consistently reported as predominant across market classes, highlighting homogeneous disease risks along the livestock supply chain. Conversely, the variable livestock management practices reported (e.g., traded species, husbandry practices, and transhumance habits) highlighted diverse potential risks for disease dissemination among market classes. Making use of readily available commercial information at livestock markets, this study describes a rapid approach for market characterization and classification. Simultaneously, this study identifies primary diseases and management practices at risk and provides the opportunity to inform evidence-based and strategic communication, surveillance and control approaches aiming at mitigating these risks for diseases dissemination through the livestock supply chain in Cameroon.

## 1. Introduction

Trade of live animals is a major component of the agricultural sector in sub-Saharan Africa (SSA) (1). Sale of livestock represents a central cash generating mechanism for the livelihoods of an important proportion of rural households in SSA (2, 3). In Cameroon, the livestock sector represents the key source of revenue to more than 30% of the rural population contributing to almost 20% of the overall agricultural gross domestic product (GDP) (4). In particular, large and small ruminants in the country are mainly commercialized in live animal markets through a consistent and robust livestock trade network (5).

Live animal markets along the livestock value chain are trading sites where various economic agents (e.g., individuals or businesses) actively interact and negotiate (3, 6). In Cameroon, supplying and demanding agents at livestock markets carry out transactions by exchanging live animals. These non-state agents (e.g., traders and intermediaries) engage in trading activities supervised by state actors (veterinary and municipal authority) (4) who routinely record cattle trade transactions (5).

Livestock markets in diverse farming systems globally, represent hotspots for the transmission and dispersal of multiple infectious diseases and can play critical roles in epidemic outbreaks (7–12). The structural characteristics of the markets, the types and flows of traded live animals, as well as the management practices at these sites, are some of the key factors contributing to the risks associated with disease spread through the livestock value chain (13, 14). In settings with scarce resources and limited data availability, improved knowledge and understanding of these factors is of particular value to better characterize market structures and the relevant risks in order to inform targeted interventions aimed at improving animal health management. Currently, there is limited knowledge and published literature on the characteristics of livestock markets in SSA (15–19). In particular, information on the epidemiology and dynamics of infectious diseases at livestock markets are still very limited (20).

In this study we analyzed two datasets collected in the Soudano-Sahelian areas of Cameroon from some of the main livestock production regions of the country, where ruminants are by far the most widespread livestock (4). Two dataset were combined: one dataset recording cattle transactions at livestock markets and a second dataset reporting questionnaire-based interviews conducted with the markets stakeholders. Based on primary data collected at livestock markets this study aimed at characterizing markets structures and major constraints for animal health in the livestock supply chain for informing targeted interventions. Trade volume, cattle price and the intensity of stakeholder attendance were used to characterize the structural and functional organization of livestock markets. This methodological approach provided a rapid and effective classification tool to guide and manage interventions to improve animal health, and to drive further studies on the livestock marketing system of the country.

The main objectives of this study were (1) to uncover the structural and functional organization of live animal markets, (2) to identify the predominant diseases as reported by livestock stakeholders and (3) to characterize the key trading and management practices at risk for disease spread. An increased knowledge and understanding of markets structure, diseases of primary concern and key management practices will provide evidence to better inform and support the Veterinary Authorities in the conception and design of targeted interventions to improve animal health management along the livestock value chain.

## 2. Materials and Methods

### 2.1. Study Site

**Figure 1:**
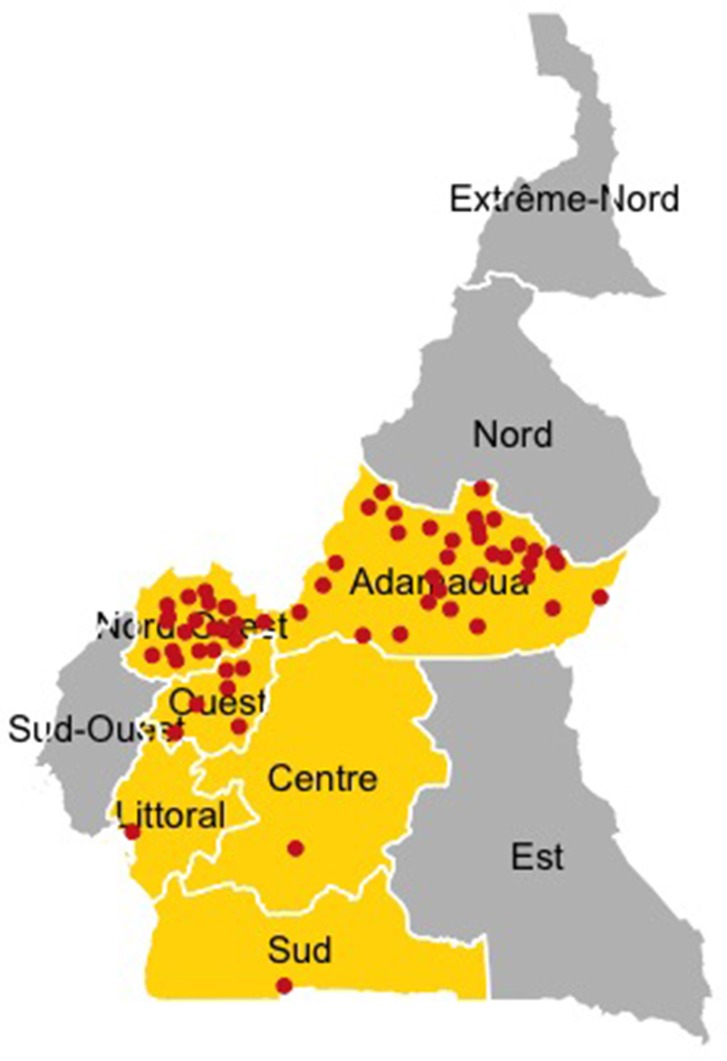
Study area and market locations. The Regions where the data collection was conducted are highlighted in yellow. The location of individual livestock markets visited between September 2014 and May 2015 are indicated by red dots.

Live animal markets in major livestock production and consumption areas of Cameroon were included in the study. These markets were located mainly across the production areas of the West, North-West and Adamawa Regions but also included markets in densely human populated and high consumption areas in the Central, Litoral and South Regions of the country (Figure 1). The Adamawa region is mainly a pastoral highland above 1,000 *m*, of approximately 64,000 *km*^2^ and considered to be the main livestock production area of Cameroon with an official animal population of about 1,900,000 head of small and large ruminants (21). The North-West region is a mountainous area covering 17,300 *km*^2^ and hosting about 4,100,000 head of small and large ruminants, while the West region is a lower lying area of 14,000 *km*^2^ with a smaller population of about 550,000 head of small and large ruminants (21). The South, Central and Littoral Regions are mainly tropical and subtropical moist broad leaf forest with a surface of, respectively, 47,191, 68,953, and 20,248 *km*^2^, and with a limited ruminant population, respectively, 80,000, 400,000, and 40,000 heads of large and small ruminants (21).

### 2.2. Data Collection

#### 2.2.1. Identification of Cattle Markets

The data collection, was carried out between September 2014 and May 2015 at all the livestock markets trading large and small ruminants and listed in the registers obtained from the relevant Regional Delegation of the Ministry of Livestock, Fisheries and Animal Industries (MINEPIA) in the West, North-West and Adamawa Regions of Cameroon (*n* = 52). Additional unlisted markets in these Regions were identified through examination of the transaction records available at the listed markets (*n* = 7) and also included (5). In addition, key livestock markets in the trading network of Cameroon (5) and located in the urban and high consumption areas of Yaoundé (Central Region) and Douala (Littoral Region), and in border area of Kye-ossi in the South Region (*n* = 3) were also included (Figure 1). Details of the livestock markets are provided in a Figure S1 and Table S1.

#### 2.2.2. Questionnaire Survey and Collection of Trading Records

At each of the 62 markets semi-structured interviews were carried out with the relevant veterinary officer, and with the other stakeholders engaged in the trading activities (herders, traders and butchers). In Cameroon, at the time of the study, details of cattle transactions carried out at markets were handwritten on paper. Simultaneously to the questionnaire-based survey, these trade records at each of these markets were scanned and all cattle transaction data recorded in these official records was extracted for a 12-month period from September 2013 to August 2014.

The administered questionnaires investigated livestock species traded at the market, diseases affecting the traded small and large ruminants and livestock management practices (e.g., number of traded or unsold cattle, the time between purchase of the animal at the market place and the introduction in the new herd, transhumance habits). The eligible population consisted of all the stakeholders conducting live animal negotiations at the market place and, therefore, the sampled population included livestock owners, traders, butchers and intermediaries. However, the context of the livestock markets, where various stakeholders are actively and intensely engaged in negotiations and transactions did not allow for selection of respondents based on representative/random sampling. The survey was, therefore, based on a convenience sampling of livestock markets stakeholders. Each questionnaire-based interview took between 5 and 10 min and was administrated either in French, English or Fufulde depending on the language of the interviewee. Details of the questionnaires used with (i) the veterinary officers and with (ii) the other livestock stakeholders are provided in the Data Sheets S2, S3, respectively.

### 2.3. Statistical Analysis

#### 2.3.1. Data Processing

From the 62 markets included in the study, all data related to cattle transactions were extracted from official records for a 12-month period, between September 2013 and August 2014. Market records were scanned using a portable device, archived as *pdf* files and then manually transcribed to an electronic database by two persons separately, and cross-checked for discrepancies. If these were identified, original scans were re-examined and data re-entered in the master database. The official markets records reported the date and price of the transaction as well as the sex, age and type of the traded cattle i.e., adult bulls, adult cows, steers, young bulls, and heifers. Cattle aged below 2 years old were considered as young animals (heifers and young bulls). Conversely, cattle older than 2 years old were considered adults (cows, bulls and steers).

#### 2.3.2. Descriptive Characterization of Commercial and Management Activities at Livestock Markets

A combination of descriptive analytic approaches was used to characterize the structure and functioning of the livestock marketing system. Temporal trends and variations in the volumes, commercial values as well as the proportions of the type of traded cattle over the 12 months of study were visualized using data from the markets transaction records. This descriptive approach was applied to the data collected through the questionnaire-based survey conducted with the market stakeholders and the relative key livestock management practices.

#### 2.3.3. Market Classes Analysis Using Hierarchical Clustering

Groups, or classes, of markets were identified using the 3 key types of information available at the livestock markets. These were (i) the size of the market, given by the total number of traded cattle over a 12-month period, (ii) the mean price of traded cattle over a 12-month period and (iii) the mean number of stakeholders attending a marketing day. The data on the size of the markets and on the mean price per head were obtained from the official documentation, and the information on the number of stakeholders attending the markets (including herders, traders and butchers) from the interviews with the veterinary officer. Clustering was performed combining the 3 information into a unique multidimensional matrix and the Euclidean distance was used to calculate the distance/similarity between the objects/markets. Hierarchical clustering analysis method (22) was applied to compute the (dis)similarity matrix between markets based on this available information. A conservative approach (Ward's method) was applied to calculate the (dis)similarity between clusters, in order to increase the confidence regarding the distance identified between the clusters, and therefore the differences between market groups, or classes (22, 23).

Data processing and analysis was carried out using scripts within the R statistical environment (24).

## 3. Results

### 3.1. General Trends of the Cattle Trade System

Records of a total of 351,345 cattle, traded between the 1st of September 2013 and August the 31st 2014, were collected within the 62 cattle markets across the study area (Figure 1 and Table 1). A total of 62 questionnaires were administrated to officials of the veterinary services and 599 questionnaires were conducted with other market stakeholders (herders, traders and butchers) (Table 1).

**Table 1:** Number of cattle markets and number of questionnaires carried out. For each market, one animal health officer representative of the veterinary services was interviewed (*N* = 62).

**Region**	**Markets number**	**Questionnaires market stakeholders**
Adamawa	31 (50.0%)	332 (55.4%)
West	6 (9.8%)	42 (7.2%)
North-West	22 (35.4%)	196 (32.7%)
Central	1 (1.6%)	10 (1.6%)
Littoral	1 (1.6%)	11 (1.8%)
South	1 (1.6%)	8 (1.3%)
Total	62 (100%)	599 (100%)

*The other stakeholders (herders, traders, and butchers) were interviewed at the market sites of each visited market in the study area. In brackets the column-wise percentage calculation*.

**Figure 2:**
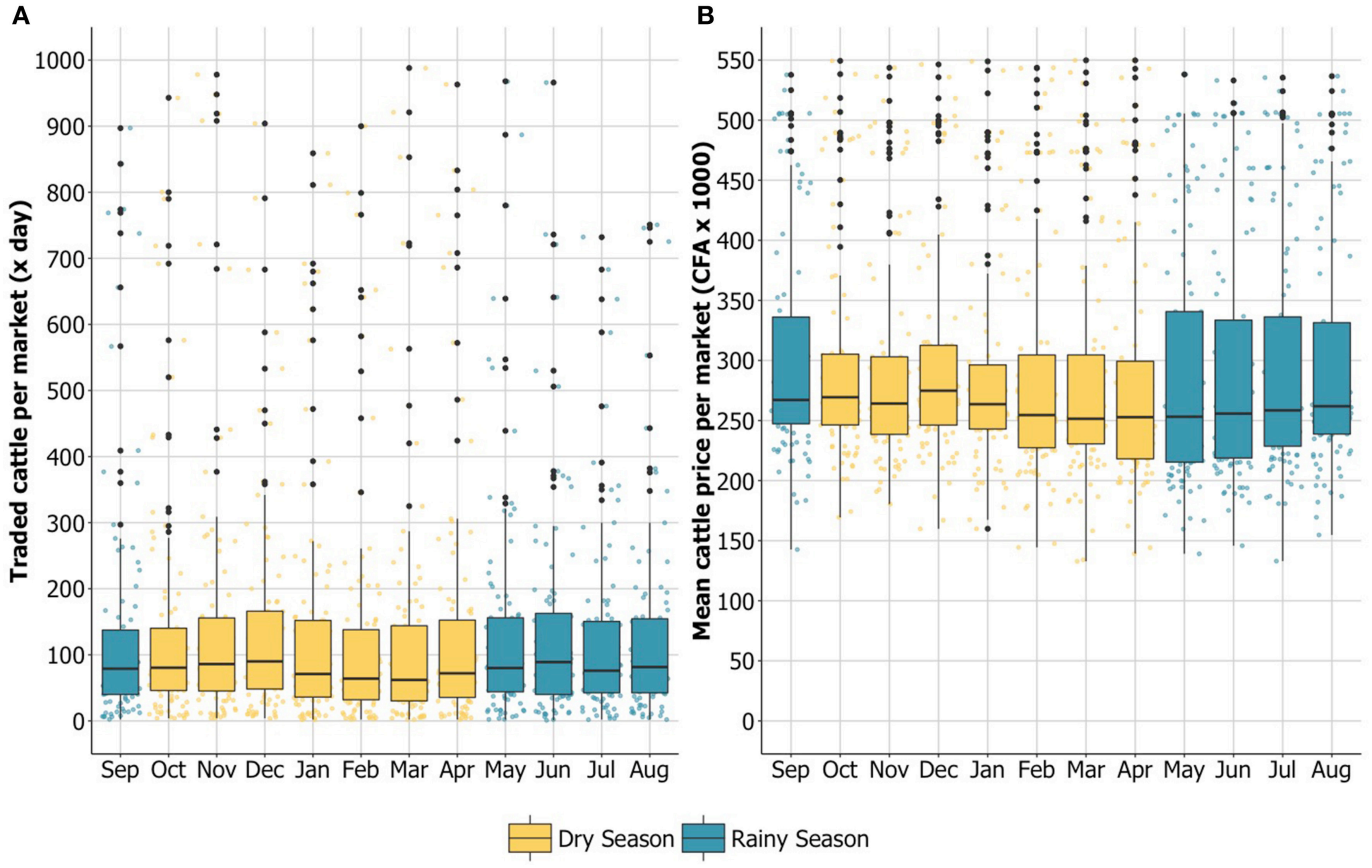
Monthly trends of traded cattle and of the mean price per head at markets in the study area. The months of the observation period between September 2013 and August 2014 are displayed on the x axis: yellow bars refer to months during the dry season and blue bars to months during the rainy season. **(A)** the y axis reports the absolute number of traded cattle per market per day of activity (range 0–1,000). **(B)** the y axis reports the mean price per head of cattle in CFA (x1000) per market (range 0–550). The upper and lower hinges correspond to the first and third quartiles (the 25th and 75th percentiles) and the line that divides the box marks the median (middle quartile).

The overall volume of traded cattle and the fluctuation of the mean price per animal across the 12-month period are shown in Figure S2. Although the number of traded cattle per month increased in the rainy season, the trading peak was registered in December (dry season) when 38,563 heads of cattle were traded. The mean price per animal also peaked in December with a mean value of 291,000 CFA for each traded cattle (Figure 2 and Figure S2). Overall, during the dry season the volume of traded cattle per month was smaller, with a minimum in February of 25,154 head of cattle (Figure S2). Despite the fluctuations in the number of monthly traded cattle, overall the range of variation of the volume of traded heads was consistent over the 12-month observation period (Figure 2A). Conversely, the mean price per head showed a higher range of variation in the months during the rainy season, compared to the dry season when mean prices at markets were more heterogenous across months (Figure 2B).

### 3.2. Markets Structural and Functional Characteristics

**Figure 3:**
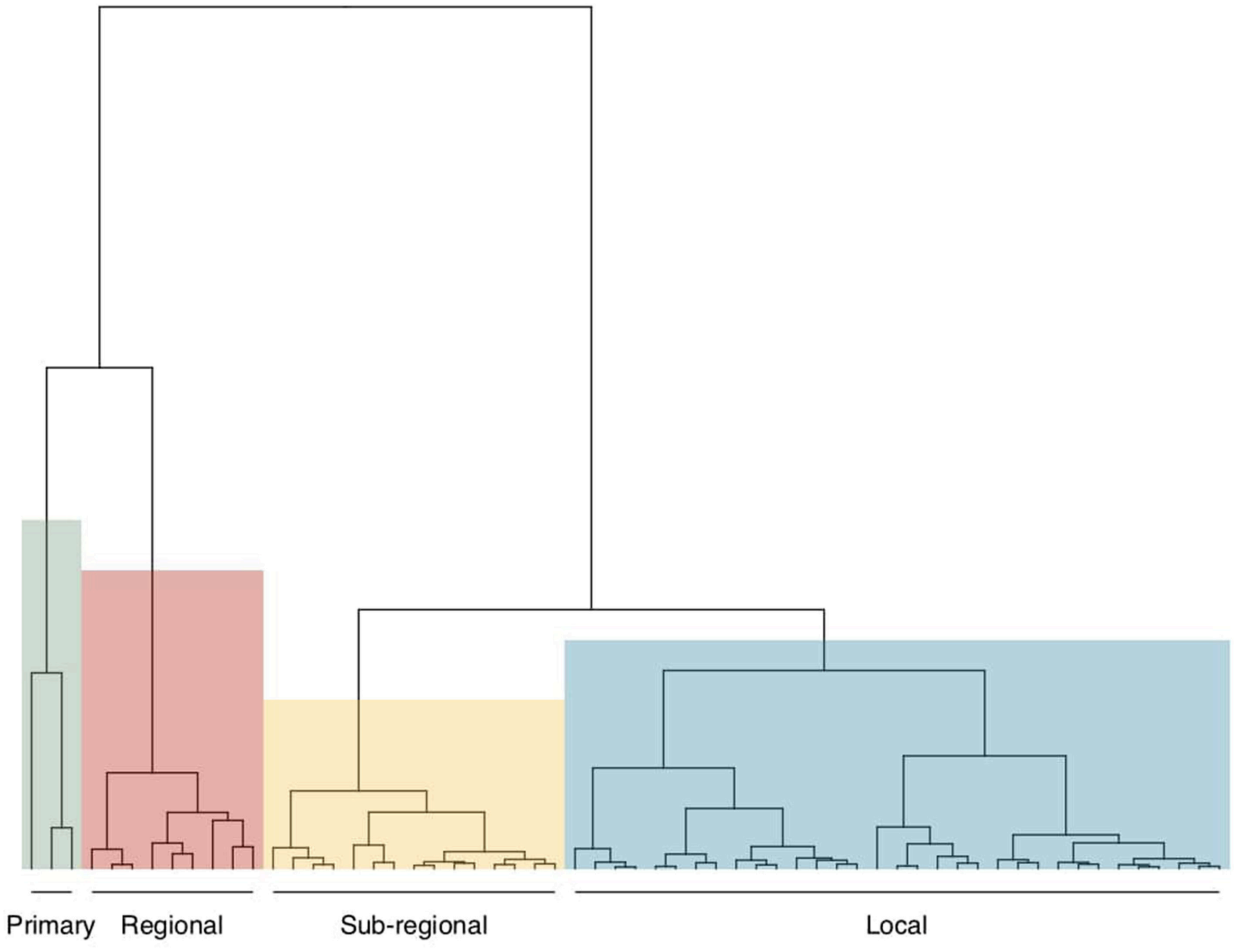
Classes of cattle markets within the study area. The four major classes of markets identified through hierarchical clustering analysis are highlighted by the four colored quadrants. The classes where named arbitrarily, although taking into consideration the visual details and qualitative and observational information gathered during the inspection at the market sites. The blue quadrant includes “local” markets, the yellow one “sub-regional” markets, the red one “regional” markets and the green one “primary” markets.

Four main sub-groups of markets were identified based on the size of the market, given by the total number of traded cattle over a 12-month period, the mean price of traded cattle over a 12-month period and the mean number of stakeholders attending a marketing day (Figure 3). The hierarchical cluster analysis identified 4 groups of markets classified in: “local” markets, “sub-regional” markets, “regional” markets and “primary” markets (Table 2). The “local” markets class included 25 markets (9 in the Adamawa Region, 11 in the North-West regionand 5 in the West Region), 21 markets were classified as “sub-regional” (13 in the Adamawa regionand 8 in the North-West Region), 13 markets as “regional” (10 in the Adamawa, 2 in the North-West and 1 in the West Region), and 3 markets as “primary” (in Southern Cameroon).

**Table 2:** Classes of livestock markets across the study area and relative characteristics.

**Market Class (interviewees)**	**Region**	**Markets Name**	**Mean cattle traded per year**	**Mean cattle price (CFA)**	**Mean number of stakeholders**
Primary (*n* = 29)	South	Kye-ossi	55,836	512,000	156
	Central	Yaounde	(9,792–87,347)	(476,000–536,000)	(95–190)
	Littoral	Douala			
Regional (*n* = 169)	Adamawa	Ngaoundere, Ngaoundal, Tello,			
		Banyo, Ngaoui, Likok, Samba			
		Mbang Foulbe, Nyambaka	7,532	242,500	154
	North-West	Bamenda, Takija	(1,395–19,498)	(202,000–311,000)	(105–223)
	West	Foumban			
Sub-Regional (*n* = 209)	Adamawa	Alme, Libong, Dir, Meiganga,			
		Margol, Mayo Baleo, Dibi,	2,720	220,400	87
		Mbanti Katarko, Kognoli, Garga,	(1,066 - 6,528)	(162,000 - 257,000)	(65 – 200)
		Mayo Darle, Martap, Galdi			
	North-West	Misaje, Binshua, Binka, Dumbu,			
		Sabongari, Esu, Wum, Bafut			
	West	–			
Local (*n* = 192)	Adamawa	Mbe, Dang, Dangfili, Djalingo,			
		Lougga, Belel, Beka Gotto,			
		Tourningal, Sambo Labo	1,529	223,300	42
	North-West	Kimbi, Subum, Ntumbaw, Mbiame, Weh,	(283–3,863)	(142,000–333,000)	(22–60)
		Wainamah, Acha Tugi, Fundong, Lassin,			
		Saje-Babungo, Tingume-Babungo, Konene			
	West	Tayandi, Bafang, Bangambi,			
		Maloua, Ngon-Kham			

*The number of respondents per markets class is reported in brackets in the first column. Mean cattle traded and mean cattle price per market refer to a 12 month period (September 2013 to August 2014). Mean number of stakeholders refer to the mean number of stakeholders participating in a marketing day (herders, traders, butchers) - In brackets, in columns 4, 5 and 6 are reported the ranges of the means of traded cattle for each class, of the mean prices of cattle per class, and of the mean number of stakeholders*.

**Figure 4:**
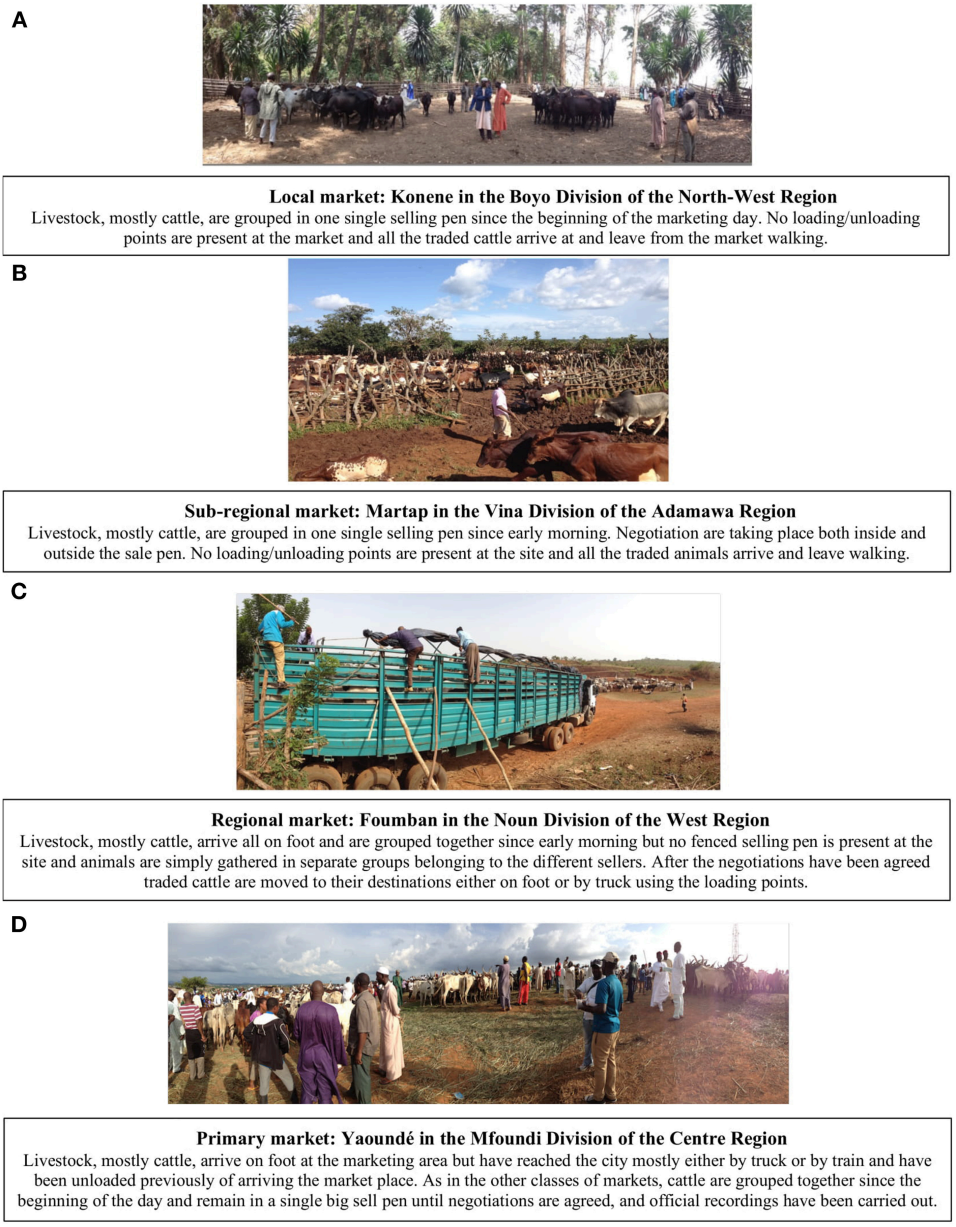
Examples of different livestock market classes across the study area. **(A)** Local market; **(B)** Sub-regional markets; **(C)** Regional market; **(D)** Primary market. Generally, the traded animals are either gathered together in one single fenced sale pen or two fenced sale pens are present, one for large ruminants and one for small ruminants, and other species if any. **(C)**, however, presents the only exception to these 2 scenarios encountered, where no fenced selling pen was present and animals were gathered in separate groups belonging to the different sellers. Pictures were taken during the data collection between September 2014 and May 2015.

**Figure 5:**
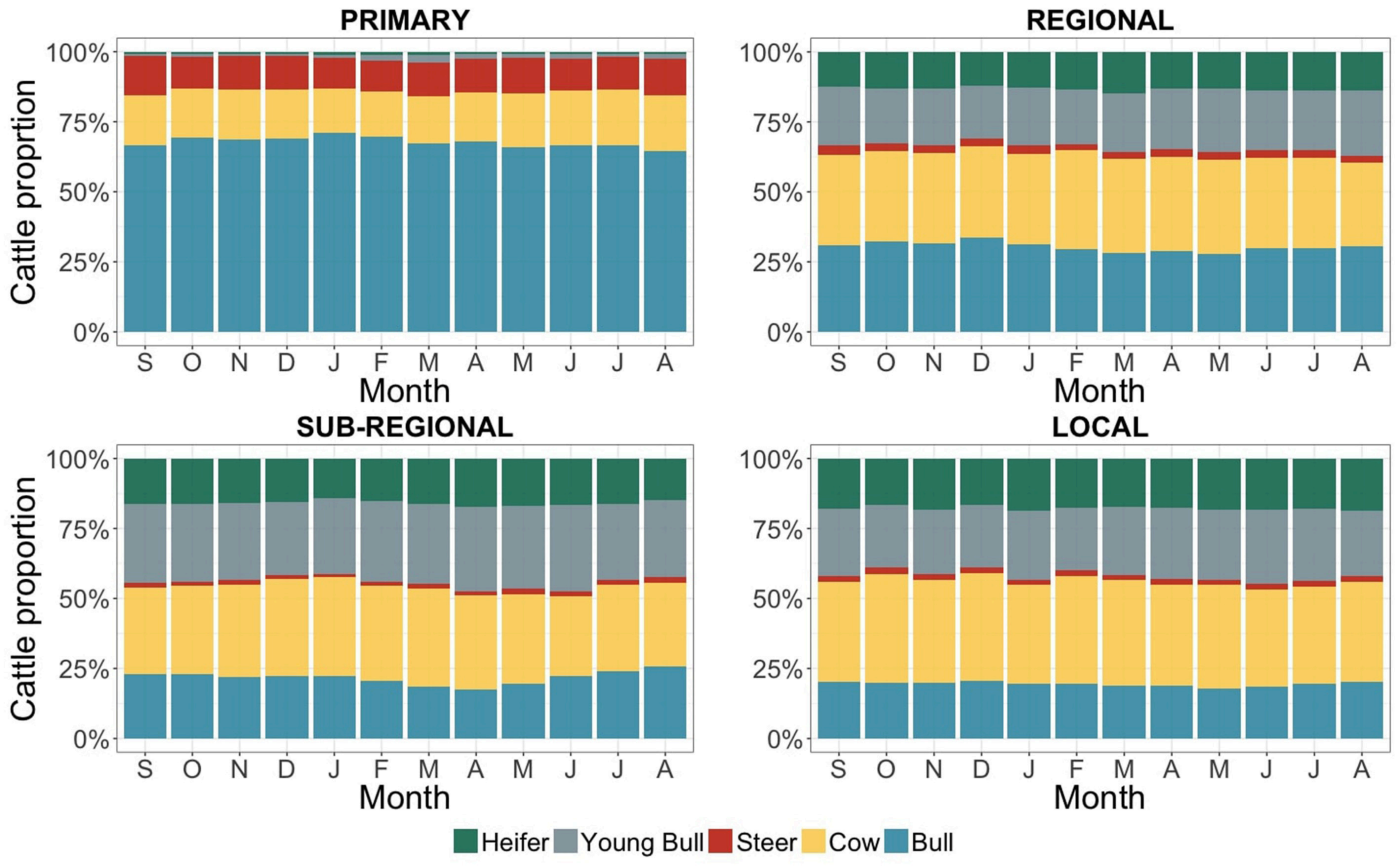
Types of cattle traded across the four classes of livestock markets in the study area. On the x axis are the months of the year from the trading records (between September 2013 and August 2014), while the y axis reports the proportions over the total number of traded cattle per month. Blue refers to adult bulls, yellow to adult cows, red to steers, gray to young bulls and green to heifers.

Examples and images of markets belonging to each of the identified classes are shown in Figure 4. There were some relevant differences in the types of traded cattle between market classes (Figure 5). At “primary” markets, adult cattle (cows, bulls or steers) were predominantly traded, accounting in total for almost 97% of the traded animals over the year. Adult cattle were also predominant at “regional” markets, where only 25–30% of the traded cattle were young animals (heifer and young bull). Young cattle (heifer or young bull) accounted for between 40 and 50% of the cattle traded in “sub-regional” or “local” markets. An interesting difference related to the sex of traded cattle. At “primary markets” a large predominance of male animals were traded across the year (ranging between 77 and 86% over the 12 months), while in the other market classes proportions of male and female cattle were approximately equivalent (around 50 male and 50% female animals). The described patterns were overall consistent across the 12-month period of observation, showing only minor monthly variations (Figure 5).

**Figure 6:**
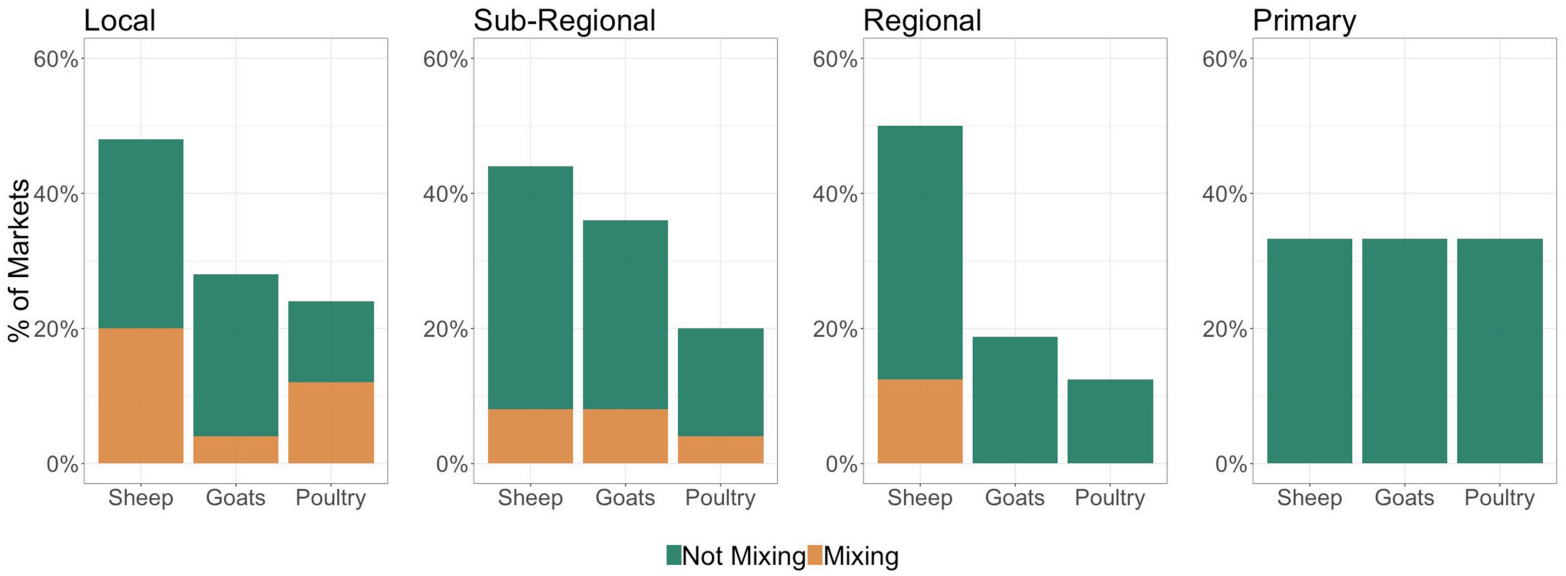
Livestock species, other than cattle, traded at the four classes of markets in the study area. The x axis reports the other livestock species traded at the different market classes (sheep, goats and poultry). On the y axis the green color refers to the percentages of markets where cattle and the other reported livestock are traded but do not mix in the sales pen. The orange color, by contrary, refers to the percentages of markets where the different species are sold in the same sales pen, therefore mixing with each other.

The presence of other livestock species varied between market classes but showed overall consistent trends across “local,” “sub-regional,” and “regional” markets (Figure 6). Sheep were consistently the most common species traded along with cattle (range: 33–50%), followed by goats (range: 18–36%) and poultry (range: 14–33%) (Figure 6). Interestingly, animals of different species were actually sold in the same sale pens, and mixed together, with the traded cattle in respectively, 31, 18, and 13% of “local,” “sub-regional,” and “regional” markets (Figure 6).

### 3.3. Disease Burden and Animal Health Conditions of the Traded Livestock

**Figure 7:**
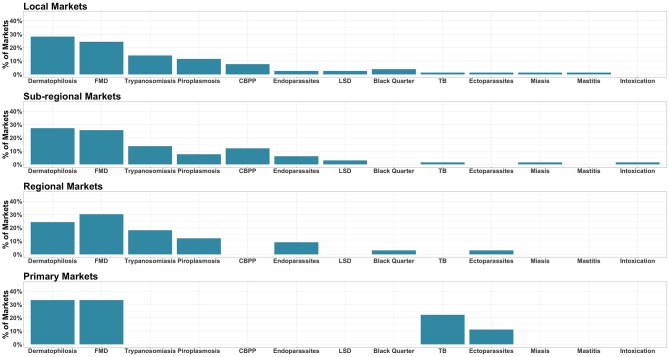
Major livestock diseases and health conditions reported at the different market classes. For each market class the reported diseases and health conditions affecting traded livestock are displayed on the x axis. The y axis indicates the percentage of markets where each specific conditions was reported as a problem affecting the animals (FMD, foot and mouth disease; CBPP, contagious bovine pleuro pneumonia; LSD, lumpy skin disease; bTB, bovine tuberculosis).

The interviewees indicated a wide range of infectious diseases and health conditions observed affecting animals traded through the marketing system. Dermatophilosis and foot and mouth disease (FMD) were consistently the most commonly reported infectious diseases across market classes (22–36% of interviewees) (Figure 7). Trypanosomiasis and piroplasmosis were also consistently reported at “regional,” “sub-regional,” and “local” markets (8–19% of interviewees). Contagious bovine pleuro-pneumonia (CBPP) was also commonly reported at these latter 2 market classes (6–12% of interviewees). Endo- and ecto-parasites were also reported as health problems by interviewees across the four market classes (2–12% of interviewees).

Overall, a number of other infectious diseases and health conditions were reported affecting traded animals, as shown in Figure 7. Nevertheless, at “local” and “sub-regional” markets the range of diseases and health conditions reported was broader, particularly in comparison with “primary” markets. Interestingly, in primary markets, bovine tuberculosis (bTB) was the third most common reported disease (22%) while being consistently one of the least reported disease in the other market classes.

### 3.4. Livestock Management Practices and Habits

**Figure 8:**
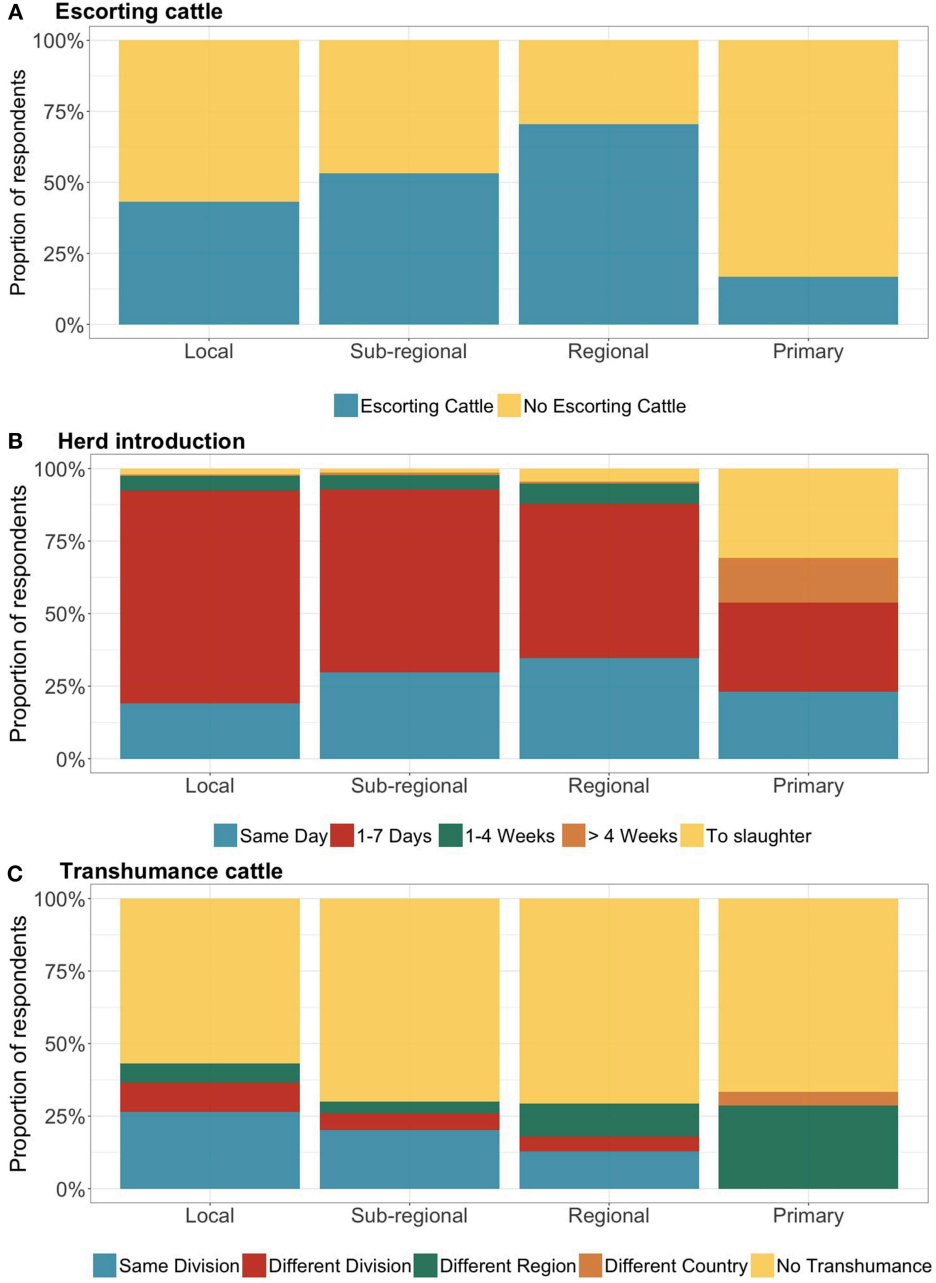
Key livestock management practices across the market classes. On the x axis respondents are grouped by market class, while the y axis is reporting the proportion of respondents per answer (*N* = 599). Interviewee on the day of the survey were asked: **(A)** Unsold cattle brought to the market reported by the interviewees selling animals on the day of the interview (*N* = 291): the yellow color refers to the proportion of respondents indicating that all the cattle brought to the market had been sold; the blue color refers to the proportion of respondents indicating that not all the cattle brought to the market had been sold. **(B)** The time of introduction in the herd of newly acquired cattle reported by the interviewees buying animals on the day of the interview (*N* = 308): on the same day (blue), within a week (red), between 1-4 weeks (green), in more than 4 weeks (orange) or sent directly to slaughter (yellow). **(C)** The transhumant practices of all the interviewees (*N* = 599): Colors in the bar chart refer to the destinations of the transhumant herds: blue within the same division, red within the same region but to another division, green to another Region, orange to another country. In yellow the proportion of respondents reporting their herds were not undertaking transhumance.

Unsold animals at livestock markets were reported by a variable proportion of interviewees which were selling animals on the day of the interview. At “sub-regional” and “regional” markets the majority of the interviewees reported not having sold all of the cattle taken to the market (53 and 69%, respectively) (Figure 8A). At “local” markets this was the case for the 43% of the interviewees and only 14% at “primary” markets.

When asked about the introduction in the herd of newly purchased animals, 27% of interviewees which were buying animals on the day of the interview reported introducing the acquired animal(s) on the same day as the purchase, and overall 87% introduced newly purchased animals into their herds within a week (Figure 8B). At “local,” “sub-regional,” and “regional” markets the proportion of introductions in < 7 days were relatively consistent (range: 92–98%) (Figure 8B).

The livestock transhumance was reported as a seasonal management practice by about 30% of all interviewees, regardless if they were selling or buying animals on the day of the interview. This proportion was relatively consistent at “sub-regional,” “regional,” and “primary” markets (range: 27–31%; Figure 8A). Nevertheless, differences were reported with regards to the destinations of the transhumance. While all the respondents at “primary” markets reported a destination in other Regions of the country, or in another country, only 14 and 3% reports, respectively, from “regional” and “sub-regional” markets indicated other Regions as the destination of transhumance. Conversely, the proportion of interviewees reporting a transhumance within the same region (either in the same or different divisions) were 18% at “regional” markets and 24% at “sub-regional” markets.

At “local” markets, transhumance as a seasonal management practice was reported by a higher proportion of interviewees (45%) (Figure 8C). The majority of these respondents (26%) reported remaining in the same administrative division, the 12% moving toward another administrative division of the same region, and only 7% to another region of Cameroon.

## 4. Discussion

Livestock markets are central aggregation events along the livestock supply chain in diverse farming systems (9–11). They represent contact points for livestock populations and stakeholders, and have key roles in the epidemiology of multiple infectious, and zoonotic diseases (13, 20, 25). Due to this strategic position within the livestock supply chain, animal markets are also ideal sites for gathering information, and communicating and mitigating disease risks and their impacts. However, epidemiologists and risk managers face major constraints in accessing relevant information at these sites.

In SSA, particularly, there are still wide knowledge gaps, and very little published literature, on the characteristics of animal markets (17–19) and of their epidemiological implications (15, 20). Based on primary data collected at livestock markets in major production and consumption zones of Cameroon we characterized the structural and functional organization of livestock markets in these areas, highlighting the major health conditions and key management practices along the livestock supply chain.

Cattle were by far the most common livestock traded at the live animal markets in the study area. Nevertheless, in about one third of the markets other livestock species were also sold (mainly small ruminants). Over the 12-month period of observation, the peak of cattle traded in the market system was recorded in December, however, the monthly trade volume was consistently larger during the rainy season (May-September). An increase in the live animals supply it is not uncommon concomitant with national and religious festivities (26, 27), and the trading peak in December (i.e., dry season) could, therefore, be directly linked to a specific rise in the demand for meat products during the end of the year's celebrations, particularly in densely human populated areas. The steady larger monthly cattle supply during the rainy season, in contrast, likely relates to a more heterogeneous combination of factors. During the dry season in fact, in SSA the scarcity of grazing areas and water sources, and the often extreme weather conditions, directly affect the health status of the herds, reducing the trading value of the animals (28), and therefore likely affecting the supply of live animals at markets. During the rainy season, conversely, the improved pastures and water availability, allow cattle to re-gaining weight and improving their general conditions and, eventually, their suitability for trade, increasing market supply and trade volumes (29).

Our study could only refer to a single year of observation and did not investigate drivers of livestock price. Nevertheless, we could observe that the period at which mean prices per head were higher, visually coincided with the period during which pasture productivity is lower (i.e., the dry season) and animal body conditions poorer. As age and gender of the traded animal are known determinants of live cattle price also in Cameroon (30), the proportional increase in traded adult animals during this period of the year could partially explain the observed higher mean prices per head. However, other factors that could not be captured in the current study might also influence these seasonal patterns of commercial values, particularly the types of cattle traded, the phenotypic and breed characteristics (31–33), as well as local cultural and traditional factors (31).

Based on the cattle trade volumes, their mean commercial values and the intensity of stakeholder attendance at markets, four main classes of markets could be identified in the study area. “Local” and “sub-regional” markets are generally located in rural or semi-rural areas and considered as collection markets where about one out of two traded cattle tend to be a young animal (heifer or young bull) and where both cattle and small ruminants are often traded. “Regional” markets are generally located in, or close to, regional administrative or commercial hubs or in towns strategically located along key transport infrastructures (e.g., tar roads or railway). Higher numbers of animals are traded, compared to “local” and “sub-regional” markets, and only about 1/3 of the cattle supply over the year are young animals (heifer and young bull). These markets also represent terminal trading points where live animals are directly sold for human consumption or further traded toward “primary” markets in Cameroon, where traded cattle are almost exclusively adult males, or toward neighboring countries (5).

The variable proportions of cattle types traded across market classes might reflect different drivers for trading live cattle. More commercially valuable adult males at “regional” and “primary” markets are more suitable for human consumption (32, 34), compared to heifers and young bulls predominantly traded at “local” and “sub-regional” markets. In pastoral areas, in particular, young animals are often traded for restocking and breeding purposes (32). Therefore, depending on the market class, diverse motivations and drivers for trading cattle might apply. At “primary” markets, direct slaughtering and exporting are important trade drivers, while at “local” and “sub-regional” markets restocking or satisfying households needs represent major trading motivations (3). Drivers of livestock commercialization are important factors for better understanding production systems as well as patterns of trade-related animal movements, and further research should focus in investigating these factors in livestock supply chains in SSA.

Animal age, while being a key driver of livestock price formation as discussed above (30, 32), it has also a central role in determining population-level patterns of infection and morbidity, particularly in endemic settings where multiple co-infections can coexist (35). In Cameroon multiple diseases are considered endemic (4) and the age of the host can influence the epidemiological implications of single or multiple co-infections (35). In the case of rapidly spreading infectious diseases, such as FMD, young animals are usually more susceptible to develop evident clinical signs and, also, more prone to virus dissemination (36). In the current study, FMD together with dermathophilosis, were the most commonly reported diseases. Although these reports are not confirmed by any diagnostic and laboratory evidence, they support previous investigations identifying cattle trade as the main route of entry and dissemination of foot and month disease virus (FMDV) in Cameroon (37).

Unfortunately, the epidemiology of zoonotic diseases in Cameroon is still largely unknown and, despite evidence of the presence in the livestock populations of diseases such as brucellosis, leptospirosis, Q fever and Rift Valley Fever (RVF) (38, 39) to our knowledge, there are no other studies addressing these gaps, particularly along the livestock supply chain. Interestingly, in the current study, bTB was reported as a common disease affecting traded cattle at “primary” markets. While reports of bTB cannot be other than a suspicion, given the diagnostic challenges in this context, the “proximity,” within the supply chain, between “primary” markets and slaughterhouses is of concern. Recently, a low level of bTB awareness was reported among pastoralists in Cameroon (40), while in contrast, a high prevalence of *Mycocterium bovis* was documented in cattle at Cameroonian slaughterhouses (41). These findings might suggest that markets stakeholders could have different levels of awareness of bTB, and perhaps generally of zoonotic diseases, across the different market classes of the livestock supply chain.

The trade volumes, their seasonal variations, the diverse livestock species, as well as the variety of infectious diseases reported to affect traded animals are key features contributing to making livestock markets hotspots for the dispersal of infectious diseases (7–11). However, a number of management practices along the livestock supply chain in Cameroon currently contribute to increasing the risks for infectious diseases dissemination. In the present study we saw above 80% of interviewees reporting a rapid introduction of newly purchased animals into herds, with about 1/3 of these being introduced on the same day as purchase. Clearly this practice facilitates the spread of pathogens and the adoption of isolation or quarantine periods before the introduction of purchased animals into their herd could help reducing this risk. In addition, a significant proportion of the cattle arriving at the markets were not eventually sold, particularly at “local,” “sub-regional,” and “regional” markets. While in some cases this surplus could be due to a lower market demand, it is important to note that a common practice among herdsmen in pastoral areas of Cameroon is to walk additional animals other than the ones for sale to the markets (4). This escorting practice makes it more practical for the herders to handle animals for sale during the trek to and from the market, particularly if they are accompanied by some elderly animals from their own herd (personal communication). By potentially increasing the intensity and rates of contacts, these management practices pose risks for the spread of pathogens along the livestock supply chain, particularly for rapidly spreading diseases, such as FMD and other transboundary animal diseases.

Improving basic management practices at herd and market levels is, therefore, a priority. At market level, despite potential infrastructural constraints, correct separation between escorting and trading animals, and between different livestock species, is a relatively simply applicable measure. Effective segregation of new animals before herd introduction is also a measure which can be easily implemented for risk mitigation at herd level, and that could be effectively communicated to livestock stakeholders attending the markets.

Seasonal transhumance is another important management practice in SSA, in particular for coping with environmental constraints (4, 42, 43), and in Cameroon cattle herds are known to travel long distances during this migration (44). In the current study, although the migratory routes and exact transhumance locations were unknown, around one third of the livestock stakeholders interviewed reported that their herds used to undertake seasonal transhumance. These reported herd movements to and from transhumant areas could contribute to explain why animal trypanosomiasis was consistently reported as the third most common disease affecting livestock at “local,” “sub-regional,” and “regional” markets, although it is generally considered a geographically limited and seasonally related disease (45). In fact, in Cameroon, animal trypanosomiasis is particularly prevalent in areas where rainfall is above 1,000 mm per year (38), which therefore offer greener pastures and greater water availability representing favorable areas for vector proliferation as well as for transhumant herds.

Although the list of reported diseases is likely not to be exhaustive of the health conditions affecting livestock in the country, to our knowledge, this is the first investigation listing and providing a quantitative assessment of predominant diseases affecting animals along the livestock supply chain in SSA. However, an obvious limitation of these specific findings is that disease reporting was only anecdotal and without any supporting diagnostic evidence. Additionally, this is not a complete representation of the livestock market system in Cameroon, particularly because we could not complete the study in the North and Extreme-North of the country. Another shortcoming of this survey was the convenient sampling, as a more robust sampling design could not be applied in this context where multiple stakeholders are hectically engaged in negotiations and transactions.

## 5. Conclusion

Animal markets have important socio-economic functions for livestock smallholders and producers, particularly in agricultural sectors with limited resources such as in SSA. Formal quantitative approaches are increasingly needed to understand the interactions between livestock production and trade systems, pathogen transmission and the wider environment. The design of cost-effective disease management strategies benefits of prior epidemiological knowledge, and this study aimed at providing the baseline for more evidence-based interventions where live animal trading constitutes a major risk for disease introduction and dissemination. Despite the strategic position of markets within the livestock supply chain, epidemiologists and risk managers face major constraints in accessing information on livestock markets characteristics, transactions, and management practices. Nevertheless, markets provide the opportunity for targeted mechanisms to manage risks associated with livestock production (e.g., demand and supply fluctuations) and animal health (e.g., spread of animals and zoonotic diseases). Based on information readily available at livestock markets in Cameroon, we proposed a rapid approach (1) to characterize major markets classes along the livestock supply chain, and we have (2) defined a relatively limited pool of infectious diseases predominantly affecting traded animals, as reported by livestock stakeholders. Furthermore, the identified (3) management practices at high risk for disease transmission, combined with the seasonal variations in trade volumes, offer the opportunity to inform evidence-based and strategic communication, surveillance and control approaches targeting the diverse market classes. These may include the establishment of a targeted surveillance system in the areas surrounding key markets, improving disease reporting by raising awareness and strengthening the reporting mechanisms, as well as improving management and biosecurity measures at the market and herd levels. Altogether, these findings also offer information to provide initial guidance for more evidence-based strategies to reduce animal and public health risks along the livestock supply chain.

## Ethics Statement

This research was authorized by the Ministry of Scientific Research and Innovation (Research permit number: 0119/MINRESI/B00/C00/C010/nye), and approved by the Cameroon Academy of Sciences (approval number 0371/CAS/PR/ES/PO). In the United Kingdom approval was given by the Veterinary Ethical Review Committee of the Royal (Dick) Veterinary School of the University of Edinburgh (approval number 28/14). All methods for data collection were performed in accordance with the relevant regulations, and in compliance with the received guidelines. Interviewers were trained to provide the information regarding the consent process to be communicated to the participants and the informed consent was obtained from all subjects. Oral consent was obtained due to the variable level of literacy of the respondents. Prior to interviewing, the study objectives, procedures and the content of the questionnaires were also explained to the participants who were made aware that they were under no obligation to participate if they did not want to. Questionnaires were treated anonymously, therefore, they were assigned an identification number relative to the market and the date of collection.

## Author Contributions

PM, TP, VT, and BB: designed the study. PM, VN, and SH: performed the field work. PM: conducted the analyses, interpreted the results and drafted the manuscript. PM, TP, IH, KM, VT, and BB: revised and reviewed the manuscript.

### Conflict of Interest Statement

The authors declare that the research was conducted in the absence of any commercial or financial relationships that could be construed as a potential conflict of interest.
