# Supplementary material for: Characterizing Livestock Markets, Primary Diseases, and Key Management Practices Along the Livestock Supply Chain in Cameroon
Source: Front Vet Sci. 2019 Apr 10;6:101. doi: 10.3389/fvets.2019.00101 (PMC6467964; doi:10.3389/fvets.2019.00101)
Supplement: Supplementary file 1 [file Data_Sheet_1.PDF]

Supplementary Material: Characterizing livestock markets, primary diseases and key management practices along the livestock supply chain in Cameroon

1 SUPPLEMENTARY TABLES AND FIGURES

1.1 Data S1: Data Collection

The proportions of markets listed within the official registers and present within the study Regions of the Adamawa, West and North-West (n=52), and of markets identified through the preliminary analysis of the official reports located within these three Regions (n=7) were overall consistent across the three Regions of the study area (Figure S1). This data collection approach enabled us to identify a total of 59 cattle markets within the Adamawa, West and North-West Regions where active data collection was carried out (Figure S1). Additionally 3 markets located in Yaounde and Douala, the two major urban centres of Cameroon, and in Kye-ossi, a border town in the South Region neighbouring with Gabon and Equatorial Guinea) were included in the study.

1.2 Figures and Tables

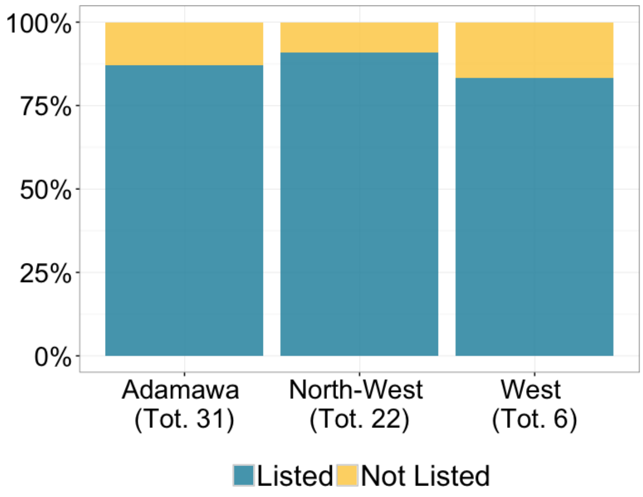

Figure S1. Proportions of markets identified through the official list and though the investigation within the three main study Regions (n=59). Blue colour refers to the proportion of the markets that were officially listed (n= 52) while the yellow colour refers to the markets that were not on this lists (n= 7).

Table S1. Names and Regions of the identified cattle markets across Central and Southern Cameroon.

Region	Markets location
**Adamawa**	Ngaoundere, Ngaoundal, Tello, Banyo, Ngaoui, Galdi, Nyambaka, Likok, Mbang Foulbe, Samba, Mayo Darle, Mbanti Katarko, Martap, Dibi, Margol, Mayo Baleo, Belel, Beka Gotto, Sambo Labo, Alme, Libong, Dir, Meiganga, Garga, Kognoli, Mbe, Dang, Dangfili, Djalingo, Tourningal, Lougga
**West**	Foumban, Tayandi, Bafang, Bangambi, Maloua, Ngon-Kham
**North-West**	Bamenda, Takija, Misaje, Binshua, Sabongari, Esu, Wum, Bafut, Kimbi, Subum, Binka, Dumbu, Ntumbaw, Tingume-Babungo, Saje-Babungo, Mbiame, Weh, Wainamah, Acha Tugi, Konene, Lassin, Fundong
**Central**	Yaoundé
**Littoral**	Douala
**South**	Kye-ossi

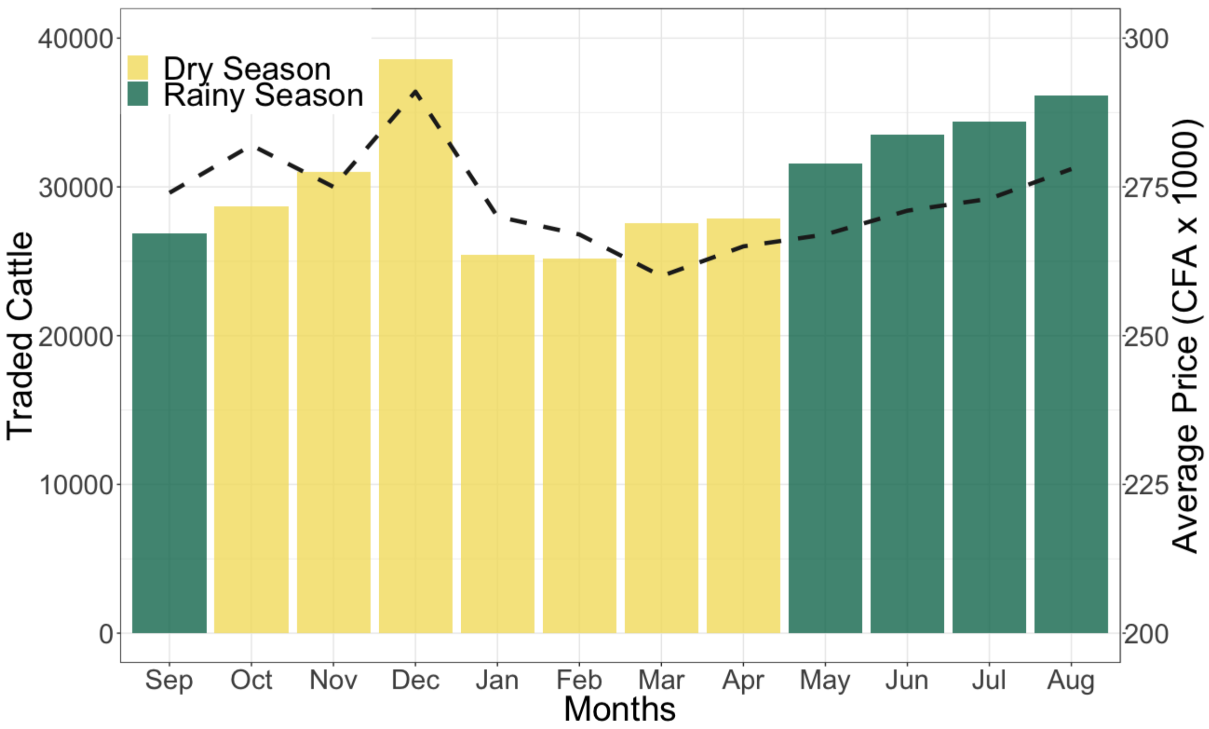

Figure S2. Monthly trends of traded cattle and of the mean price per head in the entire study area. The months of the observation period between September 2013 and August 2014 are displayed on the x axis: yellow bars refer to months during the dry season and green bars to months during the rainy season. The y axis reports the absolute number of traded cattle per month. The dashed grey line refers to the mean price per head of cattle in CFA x1000 reported on the z axis.
