# Supplementary material for: Characterizing Livestock Markets, Primary Diseases, and Key Management Practices Along the Livestock Supply Chain in Cameroon
Source: Front Vet Sci. 2019 Apr 10;6:101. doi: 10.3389/fvets.2019.00101 (PMC6467964; doi:10.3389/fvets.2019.00101)
Supplement: Supplementary file 2 [file Data_Sheet_2.PDF]

M |__|__|	|__|__|__|__|
ID of market	ID of Manager

Name of the interviewer: _______________________ **Date:** day \|__\|__\| month \|__\|__\| 201__

1. Market animal health officer information

1.1 What is your full name ? ________________________________

1.2 How long have you been working in this market? ___________________________________

1.3 How are animals mainly brought to this market? (Rank the options it in order of importance, give them the same rank to equally important).

(1) On foot	(2) Private motor vehicle	(3) Common motor vehicle	(4) Train	(5) Others

Comments:____

____

1.4 What animal species, other than cattle, are currently traded in this market? (Rank options in order of importance, give them the same rank to equally important).

(1)Sheep	(2)Goats	(3)Poultry	(4) Other

Comments:____

____

1.5 Are these different species traded in the same sale pen? Yes ☐ No ☐

1.6 How many stakeholders are attending the market today day?

	Traders	Herders	Butchers	Other
Dry Season				
Wet Season				

Other: ____

1.7 How many stakeholders are attending an average market day in the different seasons of the year?

	Traders	Herders	Butchers	Other
Dry Season				
Wet Season				

Other: ____

1.8 Market transaction costs:

Each traded animal	CFA
Buyer	
Seller	

	CFA
Access the market	
Travel Documents	

1.9 Which are the main transhumance destinations of cattle herds normally grazing in this District?

Village\Market	Subdivision	Division	Region*	N° of walking days from the market location
1				
2				
3				
4				
5				
6				

***If another country, specify here :**

____

2. Marketing Information

A – RAINY SEASON in the past year

2.1 According to your knowledge where were the cattle traded in this market COMING FROM?

Village\Market	Subdivision	Division	Region*	N° of walking days	N° of hours by vehicle
1					
2					
3					
4					
5					
6					
7					
8					
9					
10					
11					
12					
13					

***If another country, specify here :**

___

___

___

2.2 According to your knowledge where were the cattle traded in this market DIRECTED?

Village\Market	Subdivision	Division	Region*	N° of walking days	N° of hours by vehicle
1					
2					
3					
4					
5					
6					
7					
8					
9					
10					
11					
12					
13					

***If another country, specify here :**

B – DRY SEASON in the past year

THE SAME OF THE WET SEASON ☐ OR:

2.3 According to your knowledge where were the cattle traded in this market COMING FROM?

Same of wet season ☐ or:

Village\Market	Subdivision	Division	Region*	N° of walking days	N° of hours by vehicle
1					
2					
3					
4					
5					
6					
7					
8					
9					
10					
11					
12					
13					

***If another country, specify here :**

2.4 According to your knowledge where were the cattle traded DIRECTED? Same of wet season ☐ or:

Village\Market	Subdivision	Division	Region*	N° of walking days	N° of hours by vehicle
1					
2					
3					
4					
5					
6					
7					
8					
9					
10					
11					
12					
13					

***If another country, specify here :**

____

____

____

3. Animal health conditions

4.1 Could you list all the livestock diseases affecting the small and large ruminants that you have observed at this livestock market?

4.2 Could you list all the livestock diseases affecting the small and large ruminants that you have observed in your area of competence?

4.3 Could you rank the 3 primary livestock diseases or animal health conditions you have observed in this market in the past 12 months?

Disease	Livestock Species

4.4 Were these 3 livestock diseases consistently the most commonly visible at the market over the past 3 years?

We may need to contact you to clarify some of your answers. Could you please give us your mobile number? (optional)

THANK YOU FOR YOUR PARTICIPATION IN THIS SURVEY!
