# Supplementary material for: Characterizing Livestock Markets, Primary Diseases, and Key Management Practices Along the Livestock Supply Chain in Cameroon
Source: Front Vet Sci. 2019 Apr 10;6:101. doi: 10.3389/fvets.2019.00101 (PMC6467964; doi:10.3389/fvets.2019.00101)
Supplement: Supplementary file 3 [file Data_Sheet_3.PDF]

**M** |__|__|	|__¦__¦__¦__|
ID of market	ID of seller/buyer

Name of interviewer: _______________________ **Date:** day \|__¦__\| month \|__¦__\| 201__ **GPS:** _________________________

1. Stakeholder Information

1.1 a) What is your full name ? ____ **b) How old are you?** years_____

c) STAKEHOLDER STATUS : Who are you in regard to the cattle presented? :

☐ Herdsmen/owner (1) ☐ Intermediary (2) ☐ Caretaker/laborer (3) ☐ Other (4) :_____________________

d) Gender : ☐ Male ☐ Female **Education level :** Secondary ☐ Intermediate ☐ Primary ☐ None ☐

1.2 Where is the usual location of your herd.

Village_________________ Subdivision___________________ Division___________________ Region __________________

1.3 How do you bring the animals to this market or to your village?

☐ On foot (1) ☐ Private motor vehicle (2) ☐ Common motor vehicle (3) ☐ Train (4) ☐ Others (5)

Commentaires**:**____

1.4 What other animals, other than cattle, do your herd/animals usually meet on the way to the market? Tick the appropriate box:

(1) Any	(2)Sheep	(3)Goats	(4)Poultry	(5)Dogs	(6)Horses	(7)Wild animals	(8)Other

1.5 From all the cattle bought and sold over the past 12 months, how many did you buy in markets and how many outside?

	**Markets**	**Ouside Markets**
**Bought** (Num.)		
**Sold** (Num.)		

1.6 How many cattle did you sell in the past year?

	Dry Season	Rainy Season
Number of cattle		

1.7 How many cattle did you buy in the past year?

	Dry Season	Rainy Season
Number of cattle		

1.8 When you buy a new animal at the market you introduce it in your herd:

On the same day ☐ After 2-7 days ☐ 1-4 weeks ☐ more ☐ : ______________________________
You buy to slaughter ☐

1.9 Did your herd/s go on transhumance in the past 12 months? Yes ☐ No ☐

If yes: Where? ________________________________ Region: __________________ Division: __________________

If yes: When? From: __________________ To: _________________

Comments:

____

____

____

____

SECTION 2

2. A - If the respondent is buying cattle on the day of the interview

2.1 How do you bring the animals to the market or to your village?

☐ On foot (1) ☐ Private motor vehicle (2) ☐ Common motor carriers (3) ☐ Train (4) ☐ Others (5)

Comments: ____

2.2 From whom did you buy?

☐ Intermediary How many Intermediaries? ________ Where are they from? ____________________

☐ Herdsmen How many Herdsmen? ________ Where are they from? ____________________

☐ Others: Comments: ____

2.3 How many livestock did you buy today?

	Adult cows	Adult bulls	Young bulls	Young calves
**Number**				
	Goats	Sheep	Poultry	Others
**Number**				

2.4 Purpose for buying livestock today?

	**Yes**	**No**	**Unknown**
(**A**) for slaughtering	☐	☐	☐
(**B**) for breeding or restocking	☐	☐	☐
(**C**) to re-sell	☐	☐	☐

Others: ____

2.5 When are the animals that you bought today at the market going to be introduced in your herd:

On the same day ☐ After 2-7 days ☐ 1-4 weeks ☐ more ☐ : ____________________

You buy to slaughter ☐

B - If the respondent is selling cattle on the day of the interview

2.6 How many animals did you bring to the market today to sell? ____________________________

2.7 A) How many animals did you bring in Total? ____________________________

B) Only if the interviewee brought more animals than sold at the market: why you brought more animals than the ones you sold at the market?

____

____

2.8 Who did you sell to?

☐ Intermediaries : How many Intermediaries? __________ Where are they from? ____________________

☐ Herdsmen: How many Herdsmen? __________ Where are they from? ____________________

☐ Others:____

2.9 How many animals did you sell today?

	Adult cows	Adult bulls	Young bulls	Young calves
**Number**				
	Goats	Sheep	Poultry	Others
**Number**				

2.10 Purpose for selling livestock today?

	**Yes**	**No**	**Unknown**
(**A**) Earning money to buy younger animals	☐	☐	☐
(**B**) Buy family consumables, taxes, farming inputs	☐	☐	☐
(**C**) Unknown	☐	☐	☐

Others:____

THANK YOU FOR YOUR PARTICIPATION IN THIS SURVEY
